# Synergistic Effects of Separator Architecture and Electrolyte Chemistry on the Electrochemical Stability of Lithium Ion Batteries With Li–Mn Rich Cathodes and µ‐Si Anodes

**DOI:** 10.1002/advs.78391

**Published:** 2026-10-20

**Authors:** Marian Cristian Stan, Bärbel Arling, Gerrit Michael Overhoff, Kirstin Darida, Lucas Marquart, Peng Yan, Lara Kersting, Linus Voigt, Laurin Profanter, Hyung‐Tae Kim, Martin Winter, Gunther Brunklaus, Isidora‐Cekic Laskovic

**Affiliations:** ^1^ Institute of Energy Materials and Devices Helmholtz Institute Münster: Ionics in Energy Storage (IMD‐4 / HI MS) Forschungszentrum Jülich GmbH Münster Germany; ^2^ International Graduate School for Battery Chemistry, Characterization, Analysis, Recycling, and Application (BACCARA) University of Münster Münster Germany; ^3^ MEET Battery Research Center University of Münster Münster Germany; ^4^ LG Energy Solution R&D Campus Daejeon Daejeon South Korea

**Keywords:** electrospinning, Li/Mn‐rich layered oxide, lithium dendrites, micro‐crystalline silicon, N, N‐dimethylsulfamoyl fluoride, polyacrylonitrile

## Abstract

High‐voltage, high‐capacity lithium ion batteries (LIBs) combining cobalt‐free lithium manganese‐rich (LMR) cathodes with pure silicon (µ‐Si) anodes offer a promising route toward more sustainable and cost‐effective energy storage. In this study, the capacity fading behavior of LMR||µ‐Si cells is correlated with the poor wettability of the conventional polyolefin Celgard 2500 separator, which leads to non‐uniform electrolyte distribution and localized Li metal deposition on the µ‐Si surface. Consequently, cells with Celgard separators exhibit rapid degradation, with capacity roll‐over occurring after less than 75 cycles. In contrast, replacing Celgard 2500 with an electrospun polyacrylonitrile (PAN) membrane enhances cycling stability, retaining 80% of the initial capacity after approximately 230 cycles, due to improved electrolyte wetting and mechanical contact with µ‐Si anode. Further optimization of the electrolyte composition, using *N, N*‐dimethylsulfamoyl fluoride (DMFSA) as co‐solvent with fluoroethylene carbonate (FEC), extends LMR||µ‐Si cell lifetime to more than 400 cycles, regardless of the separator type. These findings highlight the synergistic influence of separator selection and electrolyte formulation in stabilizing interphases at both electrodes. A holistic materials design approach is therefore essential for advancing the performance and durability of next‐generation LMR||µ‐Si‐based LIBs cells.

## Graphical abstract

This work reveals that synergistic engineering of separator architecture and electrolyte chemistry determines the long‐term stability of LMR||µ‐Si cell chemistry. The introduction of *N, N*‐dimethylsulfamoyl fluoride (DMFSA) as a co‐solvent with fluoroethylene carbonate (FEC) promotes robust interphases on both electrodes, effectively mitigates side reactions, and extends cell lifetime.

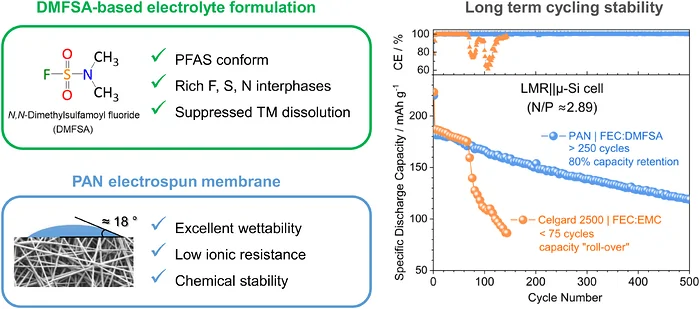

## 1 Introduction

The growing demand for high‐energy density storage, driven by the continuous advancement of consumer electronics and electric vehicles with extended driving ranges, necessitates a boost of the energy density for lithium ion batteries (LIBs) [1, 2]. Layered oxide cathode materials with specific capacities > 250 mAh g^−1^ such as lithium‐ manganese‐rich oxides (LMR), combined with silicon‐rich anodes (Si), promise an increase in energy density despite their moderate high operating voltage (≈ 3.6 V) compared to LIB chemistries with layered transition metal oxide cathodes and graphite anodes [3, 4, 5, 6]. However, the practical application of LMR cathodes is still slowed down by the many challenges that arise from their peculiar electrochemical reaction, with the surface oxygen instability as a result of Li_2_MnO_3_ activation domain at high voltage (> 4.6 V) dominating over their performance [7, 8, 9, 10]. Furthermore, to overcome the main challenges associated with the Si anode's volume variation resulting in severe morphological changes and the intrinsic oxygen release from LMR cathode active material (CAM) optimized electrolyte formulations are necessary [11, 12, 13, 14].

With its central role of “ionic bridge” between the cathode and the anode while being in contact with both electrodes and ensuring electronic insulation, the electrolyte needs to fulfill many requirements to overcome the challenges associated with these electrodes (i.e., LMR and Si) [15]. Such a challenging task requires a logical choice of the electrolyte's components (i.e., solvents, salts, and electrolyte additives) that finally through a synergistic effect stabilize the electrode|electrolyte interfaces and overcome the issues related to electrolyte decomposition and material degradation that govern the overall performance: continuous capacity and power fading, transition metal dissolution, and interfacial/interphasial instability over long‐term cycling at both electrodes [16]. To improve the long‐term cycling stability of cells with LMR cathodes, one strategy focused on interphase modification at the electrodes with electrolyte additives and specifically chosen solvents/co‐solvents [16]. For example, Zhang et al., showed that regulating the interphase chemistry with multiple electrolyte additives such as fluoroethylene carbonate (FEC) and tris(trimethylsilyl)phosphate (TMSPa) can improve the rate performance and long‐term cycling stability of LMR cathodes, achieving more than 82% capacity retention after 500 cycles at 30°C [17]. The formation of an inorganic LiF‐rich cathode electrolyte interphase (CEI) effectively protects the electrode from HF attacks, thus suppressing its structural degradation. A LiF‐rich CEI was also effectively formed using other organic solvents, such as *N, N*‐dimethyl fluorosulfonamide (DMFSA), that was reported as an electrolyte solvent for a 4 V‐class LIB [18]. In the presence of Lithium bis(trifluoromethanesulfonyl)imide (LiTFSI) as a Li‐conducting electrolyte salt, the solvent DMFSA effectively suppressed anodic Al‐current collector dissolution through its oxidative decomposition and the formation of a stable protective film containing LiF and AlF_3_. In this way, superior electrochemical performance of the LiCoO_2_||Li cells were obtained compared to an organic carbonate‐based electrolyte [18]. A LiF‐rich solid electrolyte interphase (SEI) layer was prepared by directly coating the surface of a Si anode with a slurry containing lithium polyacrylic acid (Li‐PAA) and AlF_3_. The layer protected the surface of the Si anode toward further electrolyte decomposition, stabilized the SEI, and mitigated the volume variations of the Si anodes during galvanostatic cycling, resulting in high specific capacity (> 2500 mAh g^−1^) for more than 100 cycles [19]. Man et al., modified the SEI in situ on the surface of SiO_x_ anodes by carefully selecting the pre‐lithiation agents. The SEI containing F and N‐rich components provided mechanical stability together with fast Li‐ion conductivity, resulting in impressive electrochemical performance of the LiFePO_4_||SiO_x_ cells with a capacity retention of 90.67% after 350 cycles [20]. Han et al., introduced malonic acid‐decorated fullerene (MA‐C60), that improved the stability of the interphasial layers at the LMR cathode and a Si‐containing anode by deactivating (i) the reactive oxygen species and (ii) the PF_5_ species, resulting in improved galvanostatic cycling stability [21]. These works imply the importance of controlling the CEI and SEI composition to alleviate the challenges related with the operation of LMR cathodes and Si‐based anodes in LIBs.

Furthermore, the smart selection of so‐called “inactive” materials, such as cell housing, binder, and separators can lead to an electrochemical performance improvement of the resulting cell chemistry [22, 23, 24]. The role of the separator is often overlooked, as its main task is simply to separate the cathode from the anode and to host the electrolyte. However, this basic function also requires meeting certain criteria, with chemical inertness, porosity, and mechanical stability being among the most important factors to consider when selecting an appropriate material [25, 26]. Polyolefin separators such as Celgard 2500 are commonly employed in battery cells as they are affordable, thin, and fulfill the basic tasks of a separator even though challenges such as hydro/solvophobicity, wettability, and limited porosity remain [27, 28]. Electrospinning enables to fabricate microporous membranes and with proper selection of polymeric materials, specific characteristics of the resulting membrane can be targeted [29]. Carol et al., used electrospinning to prepare a polyacrylonitrile (PAN) membrane and conducted its thorough characterization [30]. Using an electrolyte formulation composed of 1 m LiPF_6_ in a mixture of EC: DMC: DEC (1:1:1), the electrochemical performance of a LiCoO_2_ cathode with a graphite anode were investigated in the voltage range between 2.8 to 4.0 V. The obtained results indicated that when a gelated electrospun PAN membrane is used, improved properties compared to the Celgard separator could be obtained; improvements accounted on the higher porosity of the electrospun membrane that increased the electrolyte uptake and positively favored Li^+^ ion conduction [30]. Electrospinning of PAN with other components into non‐woven membranes has been used by Di Carli et al., to prepare PAN ‐polycaprolactone membranes as separators for LIBs [31]. Yu et al., showed that a composite LiCl‐PAN electrospun membrane enhanced the quality of the PAN membrane through the formation of macro‐cation complexes with the nitrile groups and the solvent molecules, which allowed the PAN molecules to be linear and orientated in a regular manner [32]. The mechanical properties of an electrospun PAN membrane were further improved by using a dispersion of nanotubes and thermoplastic polyurethane (TPU), resulting in an improvement of the electrochemical performance of lithium metal batteries (LMBs) with LiFePO_4_ using 1 m LiPF_6_ in a mixture of EC: DEC 1:1 (vol.%) as electrolyte [33]. Seo et al., developed a high‐modulus separator using the electrospinning technique based on PAN with ceramic particles [34]. The use of the high‐modulus membrane in a NMCA||µ‐Si cell setup with 1 m LiPF_6_ in FEC: EMC 3:7 (vol.%) as electrolyte showed superior electrochemical performance compared to the cells using a polyolefin Celgard 2500 separator. The authors indicated that the improved electrochemical performance can be related to the structural integrity of the high‐modulus membrane toward the large volume variations of the µ‐Si particles, whereas, in the case of the polyolefin separator, such volume variations resulted in a collapse of the separator's pores leading to poor electrochemical performance [34].

Herein, the electrochemical performance of cells using high areal capacity micro‐crystalline silicon (µ‐Si) electrodes combined with a LMR cathode (Li_1.34_Mn_0.65_Ni_0.35_O_2_) are compared between a commercial polyolefin separator (Celgard 2500) and an electrospun PAN membrane. The cells assembled with the electrospun PAN membrane demonstrated superior capacity retention, maintaining 80% after more than 232 cycles compared with fewer than 74 cycles for the cells using the polyolefin Celgard 2500 separator. Investigations revealed that the fading mechanism is affected by the properties of the separator, especially the wetting behavior. Modification of the electrolyte formulation, by replacing EMC by *N, N*‐dimethylsulfamoyl fluoride (DMFSA), resulted in enhanced cycle life and capacity retention through effective surface stabilization and improved wetting behavior. In this configuration, the effect of the separator on the cycle life of the LMR||µ‐Si cells appears, at first glance, to be less evident; however, a careful analysis of the surface morphology and composition of the two electrodes reveals that the separator still affects the interphase film formation, but in a lesser degree.

## 2 Results and Discussion

The preparation process for Celgard 2500 separator typically involves the stretching of a polypropylene (PP) thin sheet, creating nano‐pores, while the electrospun PAN membrane uses the electrospinning method of a PAN solution in DMF to fabricate the nonwoven membrane, although some additional steps, including calendaring and sintering, are necessary for the electrospun PAN membrane in order to reduce its thickness and remove the remaining solvent.

Figure 1 highlights the main differences in terms of surface morphology and wettability between the two considered separators. The morphology of the pore size can be described by a micro‐porous structure of the PAN membrane, with larger opened pores created by the nonwoven structure compared to the very narrow and nano‐porous structure of the Celgard 2500 separator (Figure 1a,b). Such changes in the porous structure of the two membranes already affects their wetting behavior, as supported by the contact angle measurements of the two separators. The Celgard 2500 separator wetted by a drop of 1 M LiPF_6_ in FEC: EMC 3:7 (vol.%) results in a higher contact angle as compared to the electrospun PAN membrane, where the liquid electrolyte spreads and permeates the separator's pores much better, resulting in a lower contact angle, i.e., 16° and 50°, for the former (Figure 1c,d). This indicates that the nature of the PP nanopores membrane is more hydrophobic toward the FEC‐based electrolyte, whereas the macropores of the electrospun PAN membrane structure are more hydrophilic in nature. The different chemistry of the two membranes, with the high electronegativity difference between the carbon and nitrogen atoms in the nitrile functional group of the electrospun PAN membrane, results in a more polar behavior of the membrane, improving the wetting behavior. Some specific properties of the two separators are listed in Table 1.

**FIGURE 1:**
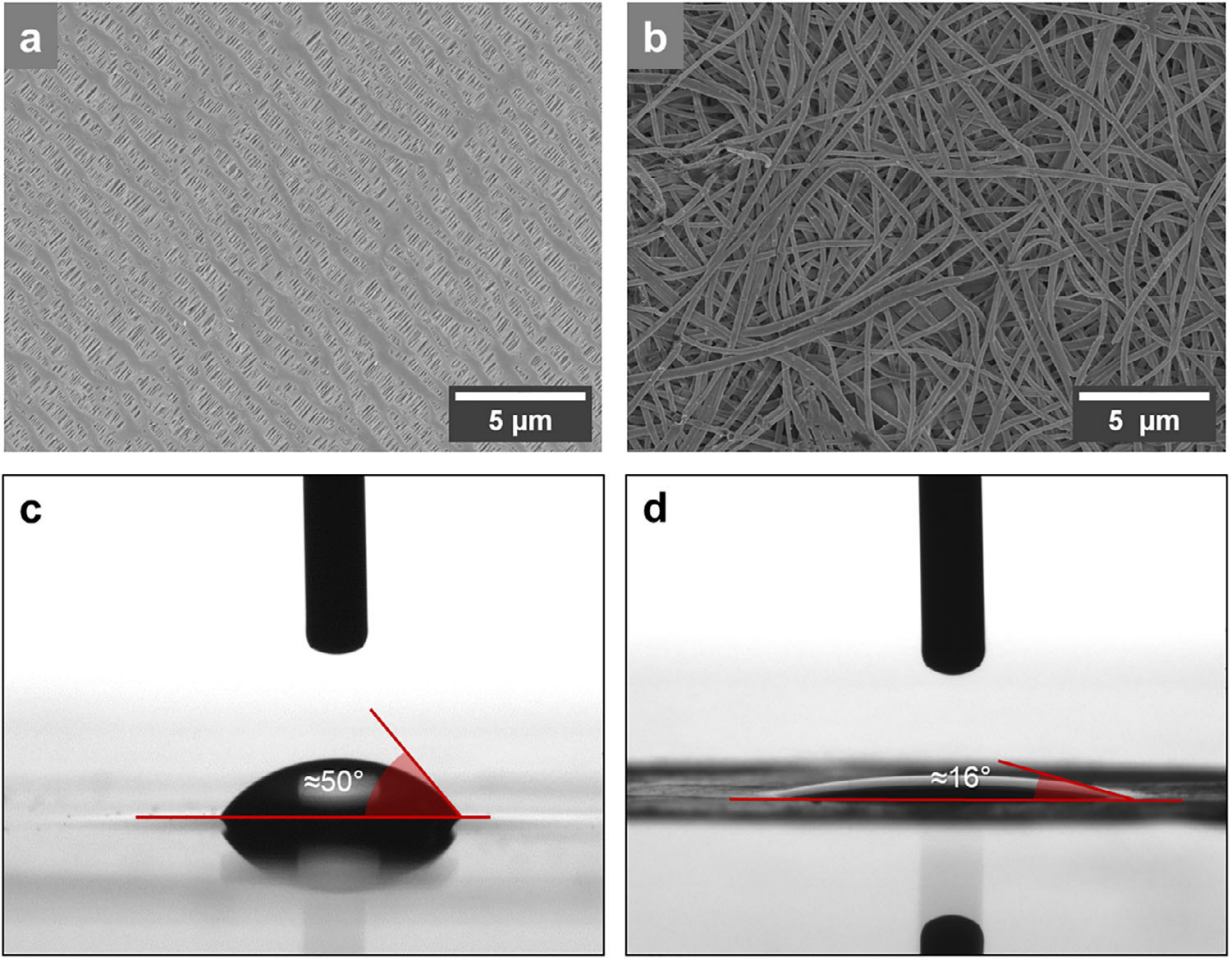
SEM images of the (a) Celgard 2500 separators and (b) electrospun PAN membrane and their corresponding (c, d) contact angle measurements using as electrolyte 1 m LiPF_6_ in FEC: EMC 3:7 (vol.%).

**TABLE 1:** Comparison between the properties of PP Celgard 2500 and PAN membranes.

Property / Feature	Celgard 2500	Electrospun PAN Membrane
Material	Polypropylene (PP) Nano porous film	Polyacrylonitrile (PAN) Nonwoven submicron fibers
Thickness / µm	22.33	21
Porosity / %	Moderate (40–45)	High (70–85)
Electrolyte uptake / %	Limited (60–80)	Very high (250–400)
Wettability	Poor – nonpolar surface	Excellent – polar nitrile groups
Ionic conductivity^a^ / mS cm^−1^	Low (0.313)	Medium (0.817)
Thermal stability / °C [35, 36]	Shrinks > 120 Melts ≈ 165	Low shrinkage Stable up to 200–250
Contact angle / °	High (≈ 50)	Low (≈ 16)
MacMullin number (N_M_)^b^	High (≈ 23.3)	Moderate (≈ 9.1)

^a^ with 1 m LiPF_6_ in FEC:EMC 3:7 (vol. %)

^b^ Ionic conductivity of the electrolyte at 20°C is 7.46 mS·cm^−1^

Among the most valuable membrane properties with respect to their applicability in energy storage and conversion devices is their effect on the ionic conductivity of the electrolyte formulation once the electrolyte is added to a cell. A modification of the ionic transport of a separator property can affect the delivered electrochemical performance, resulting in a limitation of high‐rate and long‐term performance capabilities. Such changes can be qualitatively indicated by the MacMullin number, which measures a change in the ionic conductivity of a liquid electrolyte compared to the ionic conductivity of a separator sheet wetted with electrolyte. Table 1 provides the MacMullin number measured for the Celgard 2500 separator and the electrospun PAN membrane measured by means of impedance measurements (Figure S1 and Table S1). In an ideal behavior, the addition of the electrolyte to the separator would not change the ionic conductivity, resulting in a MacMullin number of 1 [37]. A larger value is obtained for the Celgard 2500 separator compared to the electrospun PAN membrane, implying that the porosity and the tortuosity of the two separators play a role in the transport properties. The higher value could also indicate that lower electrochemical performances will be expected for the Celgard 2500 separator as compared to the electrospun PAN membrane, as will be later exemplified.

Recently, the use of µ‐Si active material has gained more attention because it enables active material loadings of up to 80 wt.% when the lithiation degree of the µ‐Si particles is controlled (via N/P capacity ratio adjustment) due to the reduced surface area resulting in less side reactions [38]. Improvement of the electrochemical performance of full cells requires a fundamental understanding of the interplay between the cell components, where both inactive and active materials play important roles. The role of the former is highlighted by the charge–discharge properties of the LMR||µ‐Si cells, as displayed by the results in Figure 2.

**FIGURE 2:**
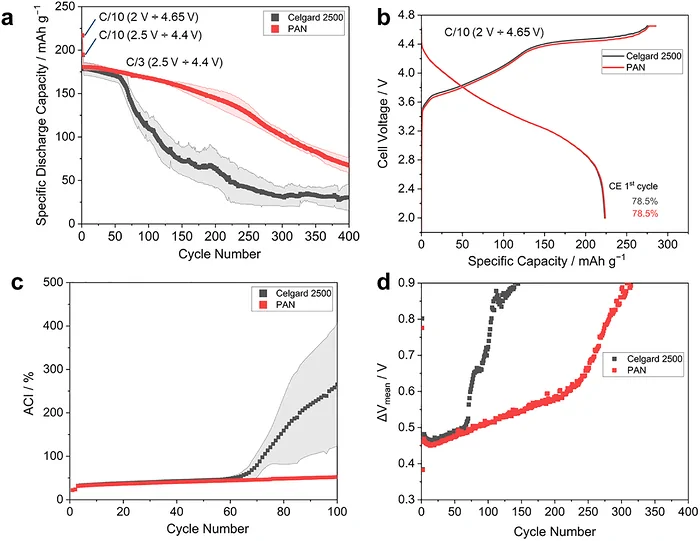
(a) Specific discharge capacities vs. cycle number of the LMR||µ‐Si cells containing 1 m LiPF_6_ in FEC: EMC 3:7 (vol.%) as electrolyte. (b) Comparison of the cell voltage profiles during the formation cycle. (c) Evolution of ACIs calculated from the CEs and (d) voltage hystereses calculated from the difference between the mean charge voltage and mean discharge voltage of the LMR||µ‐Si cells.

The electrochemical performance of the LMR||µ‐Si cells with a conventional polyolefin Celgard 2500 separator and the electrospun PAN membrane in Figure 2a shows a large difference in the cycle life even though both cells show a similar initial electrochemical behavior with identical specific discharge capacities (Figure S2). After 60 cycles, the LMR||µ‐Si cells containing the polyolefin Celgard 2500 separator reach a point where the capacity decreases drastically, i.e., “roll‐over” [39]. On the other side, when the electrospun PAN membrane is used as a separator within the same cell chemistry and with the same liquid electrolyte and amount (35 µl), a stable specific discharge capacity is observed for more than 244 cycles, before a more steeper capacity decay (possibly the “roll‐over” behavior) takes place. This accounts for more than 350% increase of the cycle life duration of the LMR||µ‐Si cells using an electrospun PAN membrane as compared to the polyolefin Celgard 2500 separator. The results indicate that the observed electrochemical behavior before the “roll‐over” effect is identical for the cells assembled with the Celgard 2500 separator and the electrospun PAN membrane, without the presence of any additional reactions or difference in the voltage hysteresis, as it can be observed from the voltage curves during the first activation cycle (Figure 2b). Furthermore, in the first 60 cycles, the accumulated Coulombic inefficiency (ACI) calculated from the Coulombic efficiency (CE) of each cycle, shows little to no difference between the two cell configurations (Figure 2c). Moreover, the data also indicate that the slopes are similar, suggesting that the side reaction rates can be considered comparable despite the different functional groups of the two separators. Here, the intensity of the effect related to the electrode|separator interaction, especially of the electrospun PAN membrane to improve the electrolyte wettability, and thus facilitate enhanced Li^+^ ion transport, is most likely overlapping by the electrochemical processes occurring at both active materials. This is further supported by the fact that the variation of the voltage difference between the mean charge and the mean discharge voltage does not indicate any difference (Figure 2d). A possible difference in the interfacial behavior between the two cell configurations would be expected, since the nitrile functional group (─C≡N) can play a role in the formation of the SEI and CEI layers. This will also slightly modify the composition of these films as compared to the cell configuration using the polyolefin Celgard 2500 separator.

The reason for the “roll‐over” failure can be accounted on various processes, such as the loss of lithium inventory (LLI) within the CAM, increased cell resistance due to either electrolyte or cathode degradation upon extended galvanostatic cycling [39, 40, 41]. It is worth mentioning that all considered cells have a N/P capacity ratio of 2.27, which should prevent Li metal plating at the µ‐Si anode since the additional capacity of the µ‐Si electrode prevents a decrease of the negative electrode potential below 0.1 V vs. Li|Li^+^. The PAN membrane exhibits higher wettability, while the presence of nitrile functional groups with an affinity toward Li^+^ ions, results in a more uniform and efficient Li^+^ ion transport and effective interphase formation at both electrodes. As a result, overall parasitic reactions are reduced, and the rapid capacity fade associated with the “roll‐over” failure is delayed to a higher cycle number (i.e., the 244^th^ cycle).

Further electrochemical investigations carried out in 3‐electrode cells, with metallic Li as reference electrode and using the similar constant current and constant voltage cycling (CCCV) procedure carried out at C/10, indicated that in the case of the LMR||µ‐Si cells assembled with Celgard 2500 separator, a slippage of the negative electrode potential toward lower values takes place with increasing cycle number (Figure 3).

**FIGURE 3:**
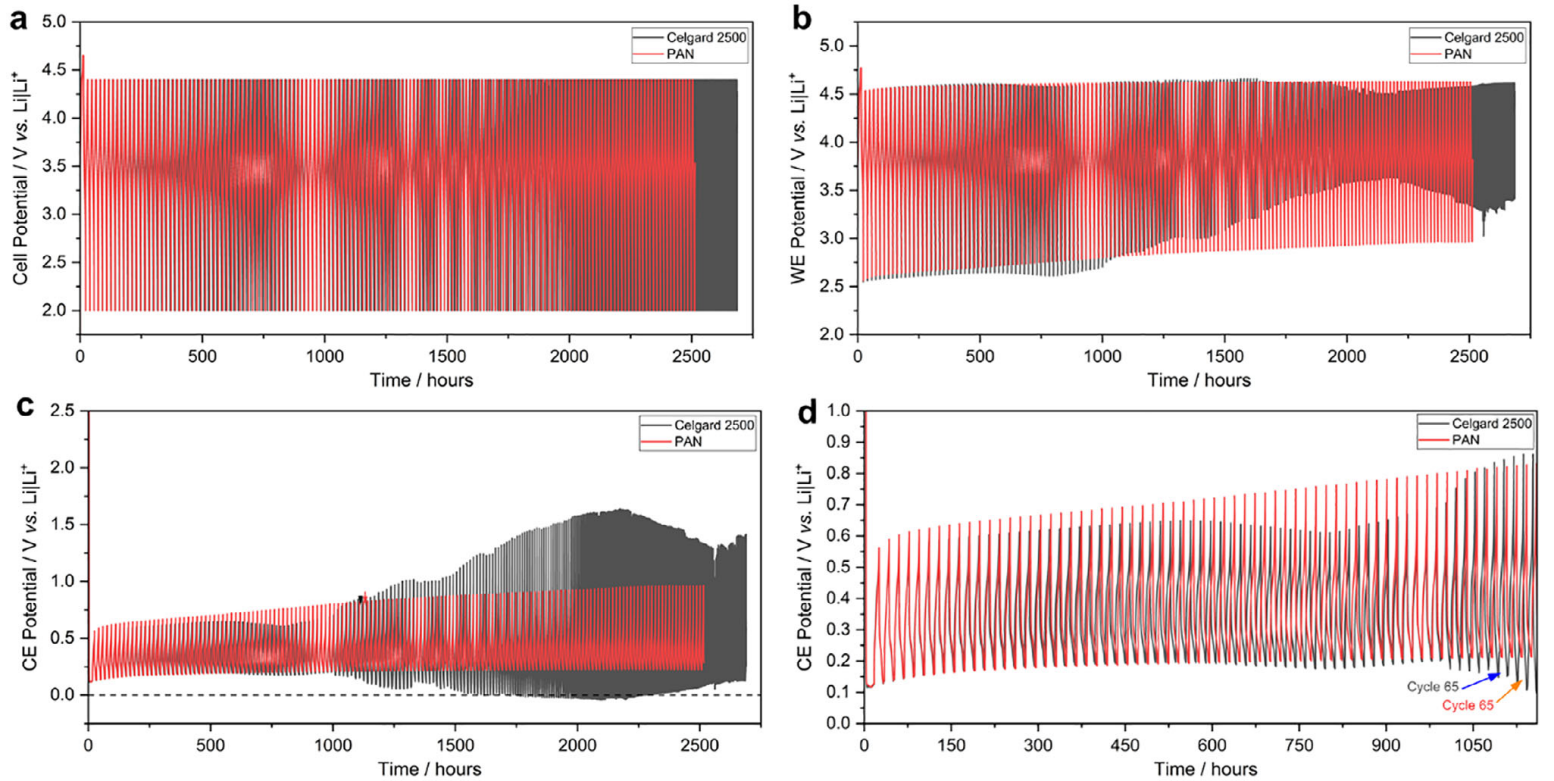
Evolution of the (a) cell potential, (b) LMR potential and (c) µ‐Si potential during the charge and discharge process of the 3‐electrode LMR||µ‐Si cells configurations with polyolefin Celgard 2500 separator and electrospun PAN membrane with a C‐rate of C/10 and 1 m LiPF_6_ in FEC: EMC 3:7 (vol.%) as electrolyte. (d) Zoom in of the anode potential evolution within the early 65 cycles.

The LMR||µ‐Si 3‐electrode cells with Celgard 2500 separator and PAN membrane, deliver, during the first ≈ 50^th^ cycles comparable specific capacities with an average ≈ 224 mAh g^−1^ in the 30^th^ cycle (Figure S3). However, despite the fact that the cell voltage is maintained between the voltage limits, i.e., 2.0 – 4.4 V during charge/discharge cycling (Figure 3a), the electrochemical performance deterioration for the cells with Celgard 2500 separator after 60 cycles can be seen in the potential profiles (Figure 3b,c). Comparing the potential profiles of the µ‐Si anodes of both cells in the 61^th^ cycle (Figure 3d), it can be observed that during the charge reaction (i.e., lithium uptake by the anode material), a drift toward lower potentials occurs for the cell with Celgard 2500 while the potential of the µ‐Si electrode from the cell using PAN membrane remains at a value ≈ 0.213 V vs. Li|Li^+^ without any changes. These changes in the potential at the µ‐Si electrode are further mirrored in the potential evolution of the LMR cathode, for the cell with the polyolefin Celgard 2500 separator, while no changes are recorded for the cells with electrospun PAN membrane (Figure 3b). Following these observations, *post‐mortem* SEM‐EDX analysis of the harvested µ‐Si electrodes (> 2500 h, Figure 3a) were carried out for the washed µ‐Si electrodes (Figure 4). The inset in Figure 4a,b presents the changes of the µ‐Si electrode's mechanical integrity with some material remaining on the Celgard 2500 separator and some gray spots, whereas no gray spots are observable on the µ‐Si electrode harvested from the cell with the electrospun PAN membrane. These differences are evident despite the fact that the same electrolyte formulation and volume were used for both separators.

**FIGURE 4:**
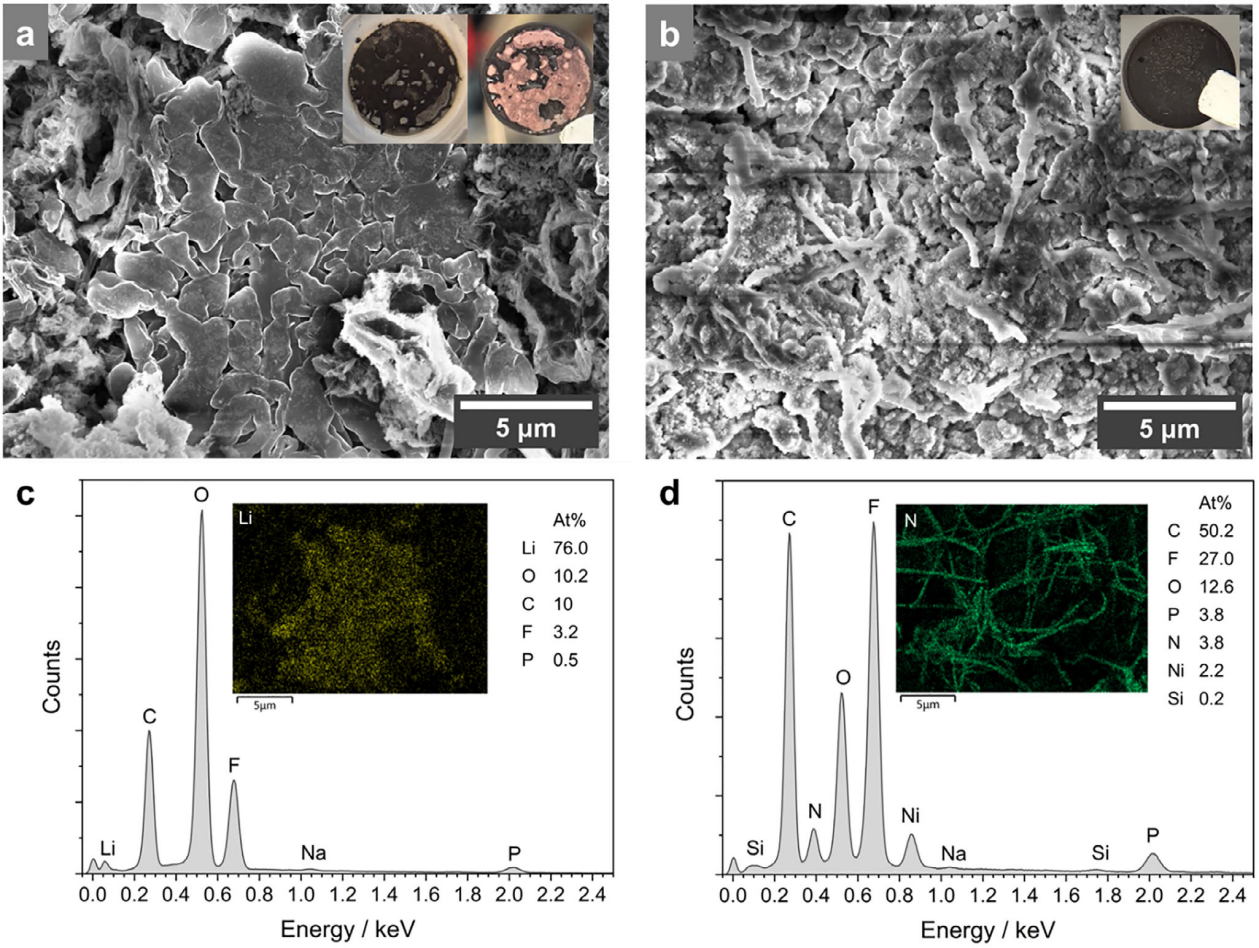
SEM images of the harvested µ‐Si electrodes from 3‐electrode LMR||µ‐Si cells with (a) Celgard 2500 separator and (b) electrospun PAN membrane. EDX of the corresponding areas indicating (c) the presence of metallic lithium in the spectrum and Li mapping image (inset) with Celgard 2500 separator and (d) the distribution of PAN fibers and N mapping image (inset) from the PAN membrane containing cells. The EDX elemental maps were selected to identify the characteristic surface species observed on each electrode (Li for the Celgard‐based cell and N for the PAN‐based cell) and are not intended as a quantitative comparison of elemental distributions.

The potential drift observed for the µ‐Si anode (Figure 3c,d) cells results in the formation of metallic Li deposits as those shown in the SEM images in Figure 4a. Such morphological structures were easily found on the surface, as well as in the bulk of the harvested µ‐Si electrode from the LMR||µ‐Si cell assembled with the Celgard 2500 separator. On the other side, no such morphological structures were observed on the surface or bulk of the harvested µ‐Si electrode from the LMR||µ‐Si cell assembled with an electrospun PAN membrane (Figure 4b). Furthermore, when comparing the morphology of the µ‐Si particles harvested from both cells, no indication of changes due to the different separators was found. Furthermore, the elemental analysis carried out for these electrodes via EDX measurements at 5 kV using a windowless EDX detector confirmed the presence of Li metal deposits on the surface of the µ‐Si electrodes harvested from the LMR||µ‐Si cells assembled with Celgard 2500 separator (Figure 4c and inset with Li mapping). The presence of metallic Li deposits, i.e., lithium metal dendrites, were also observed by SEM and confirmed by Li‐EDX on the surface of the µ‐Si electrodes harvested from the 2 electrode cells setups with Celgard 2500 separator at end of life (EOL, 1000 cycles), as presented in Figures S4 and S5. In Figure 4d, the surface analysis of the µ‐Si electrodes harvested from the cells using an electrospun PAN membrane mainly indicate elements (carbon C, oxygen O, fluorine F, phosphorus P) that are characteristic to insoluble products of the electrolyte decomposition. Since the surface features observed in the SEM image could potentially be misinterpreted as lithium deposits based solely on their morphology, the inset in Figure 4d shows the EDX elemental mapping of nitrogen (N), confirming that these features originate from residual PAN nanofibers rather than metallic lithium deposition. The formation of Li metal deposits on anode materials, such as graphite and silicon, is closely related to the local deterioration of the transport properties of Li^+^ ions, due to either surface passivation, electrolyte drying, or deposition of transition metals as a result of the cross‐talk process [42, 43]. The evolution of the Ohmic drop (i.e., cell resistance) measured from the charge and discharge pulses and the relaxation process of a LMR||µ‐Si in a three electrode setup using a GITT method did not show any relation during the initial two cycles (Figure S6), despite the differences observed with the MacMullin number. Seo et al., highlighted that the mechanical properties of the separator are of crucial importance for electrochemical performance, where the compressibility of separators during lithium uptake by µ‐Si particles was at the origin of the observed failure mechanism, as a result of the separator's pore collapse [34]. To further investigate the observed behavior, post‐mortem SEM analyses of the harvested Celgard 2500 separator were performed after 152 cycles and are included in Figures S7–S9. In contrary to the mechanism proposed by Seo et al., [34], no clear separator's pore collapse was identified (Figure S7); however, the formation of Li metal dendrites observed on the µ‐Si electrodes harvested from cells employing Celgard 2500 separator could be associated with local deterioration of the separator transport properties. This arises from extensive electrolyte decomposition resulting in the presence of adherent surface deposits and detached µ‐Si particles, indicating intimate mechanical contact between the expanding µ‐Si electrode and the separator during prolonged cycling (Figure S8). Furthermore, the surface of the µ‐Si electrode remained covered by detached interphase products transferred from the separator's surface (Figure S9). Therefore, the formation of Li metal dendrites on the surface of the µ‐Si electrodes harvested from the cells using a Celgard 2500 separator could result from local deterioration of the transport properties of the separator, since similar N/P capacity ratios were used in the present work compared to the work reported by Seo et al., (2.27 compared to 1.88). In the case of the electrospun PAN membrane, the non‐woven PAN nanofibers, besides their good wetting properties, can freely redistribute and entangle the µ‐Si particles during the volume variation (“silicon electrode breathing”) as a result of lithium uptake (charge) and lithium release (discharge) process, thus maintaining an intimate contact with the surface of the electrodes (Figure 4b) avoiding the formation of local uncontacted spots that could initiate the deposition of metallic lithium.

To further elucidate the role of separator–electrolyte synergy beyond the FEC:EMC‐based electrolyte, a second electrolyte formulation was employed with both Celgard 2500 separator and electrospun PAN membrane. *N, N*‐dimethylsulfamoyl fluoride (DMFSA) has been reported as a solvent for a “full fluorosulfonyl” electrolyte for LMBs, however, its behavior within the LMR||µ‐Si cell chemistry has not been investigated so far, despite the fluorosulfonyl imide group compatibility with Si‐based anode materials and its relatively high oxidative stability [44, 45]. Figure 5 presents an overview of the electrochemical investigations carried out in LMR||µ‐Si cells containing 1 m LiPF_6_ in FEC:DMFSA 3:7 (vol. %) as electrolyte.

**FIGURE 5:**
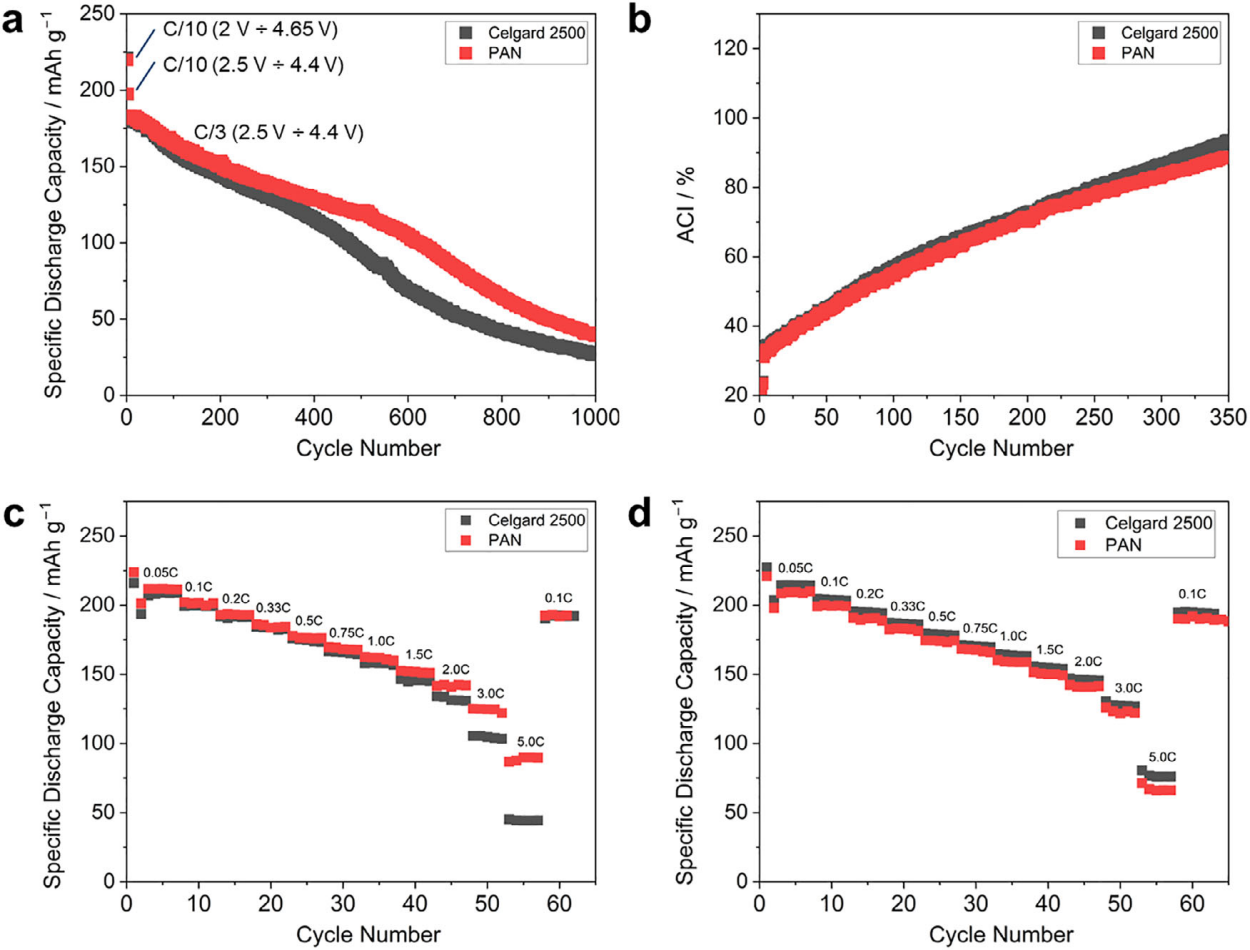
Comparison of the electrochemical performance of LMR||µ‐Si cells assembled with Celgard 2500 separator and electrospun PAN membrane showing (a) specific discharge capacity evolution and (b) calculated ACI with 1 m LiPF_6_ in FEC: DMFSA 3:7 (vol.%) (DMFSA: FEC). Effect of the separator on the delivered specific discharge capacity during the C‐rate test using (c) 1 m LiPF_6_ in FEC: EMC 3:7 (vol.%) compared to (d) 1 m LiPF_6_ in FEC: DMFSA 3:7 (vol.%).

The modification of the electrolyte formulation results in a substantial improvement of the electrochemical performance (Figure 5a), with less capacity fading compared to the cycling performance of cells with FEC: EMC‐based electrolyte (Figure S2). After 400 cycles both cells still deliver a specific discharge capacity of ≈ 116 mAh g^−1^ and ≈ 129 mAh g^−1^, that corresponds to a capacity retention calculated from the third cycle on (at C/3), of 64% for the cell with Celgard 2500 separator and 70% for the cell with the electrospun PAN membrane, respectively. Furthermore, the amount of irreversible capacity accumulated with each cycle (Figure 5b) is comparable for both cells using Celgard 2500 and the electrospun PAN membrane. Compared to the ACI calculated from the CE of the LMR||µ‐Si cells assembled with FEC: EMC‐based electrolyte and electrospun PAN membrane for the initial 200 cycles, small differences are also observed (Figure S10). The obtained results indicate that the electrochemical performance of the cells with FEC: DMFSA as electrolyte formulation are not any more strongly affected by the nature of the separator, as compared to the specific discharge capacity values presented in Figure 2a. The C‐rate capabilities of the two cells (Figure 5c,d) further indicate that the role of the electrolyte and the separator is crucial for the observed electrochemical performance. The results indicate that until a C‐rate of 1.0 C, the cells with FEC: EMC‐based electrolyte provide a comparable specific discharge capacity, whereas upon increasing the C‐rate the cells assembled with Celgard 2500 deliver a lower specific discharge capacity compared to the electrospun PAN counterparts. However, when the C‐rate is set back to 0.1C, both cells deliver similar specific discharge capacities (Figure 5c). This indicates that the local changes of the electrolyte distribution within the porous separator during the high C‐rates, affects the electrochemical behavior, however, no differences in the morphology of the electrode active materials were noticed after the C‐rate investigations (Figure S11). However, the wetting properties of the two separators with the FEC: DMFSA‐based electrolyte (Figure S9a,b) indicate a similar behavior compared to the FEC: EMC‐based electrolyte. Furthermore, the value of the MacMullin number is much more improved for the Celgard separator (Figure S12c and Table S2), an effect that is likely reflected also in the resulted electrochemical performance. On the other side, in Figure 5d the results of the C‐rate investigations in the presence of FEC: DMFSA‐based electrolyte with the two separators do not indicate substantial differences, results that are in accordance with the electrochemical performance presented in Figure 5a,b.

To gain insights into the changes occurring at the surface of these electrodes, the morphology evolution and the interphase composition variation of the µ‐Si electrodes harvested from the LMR||µ‐Si cells assembled with the two electrolytes and the Celgard 2500 separator after 50 cycles were further investigated (Figure 6).

**FIGURE 6:**
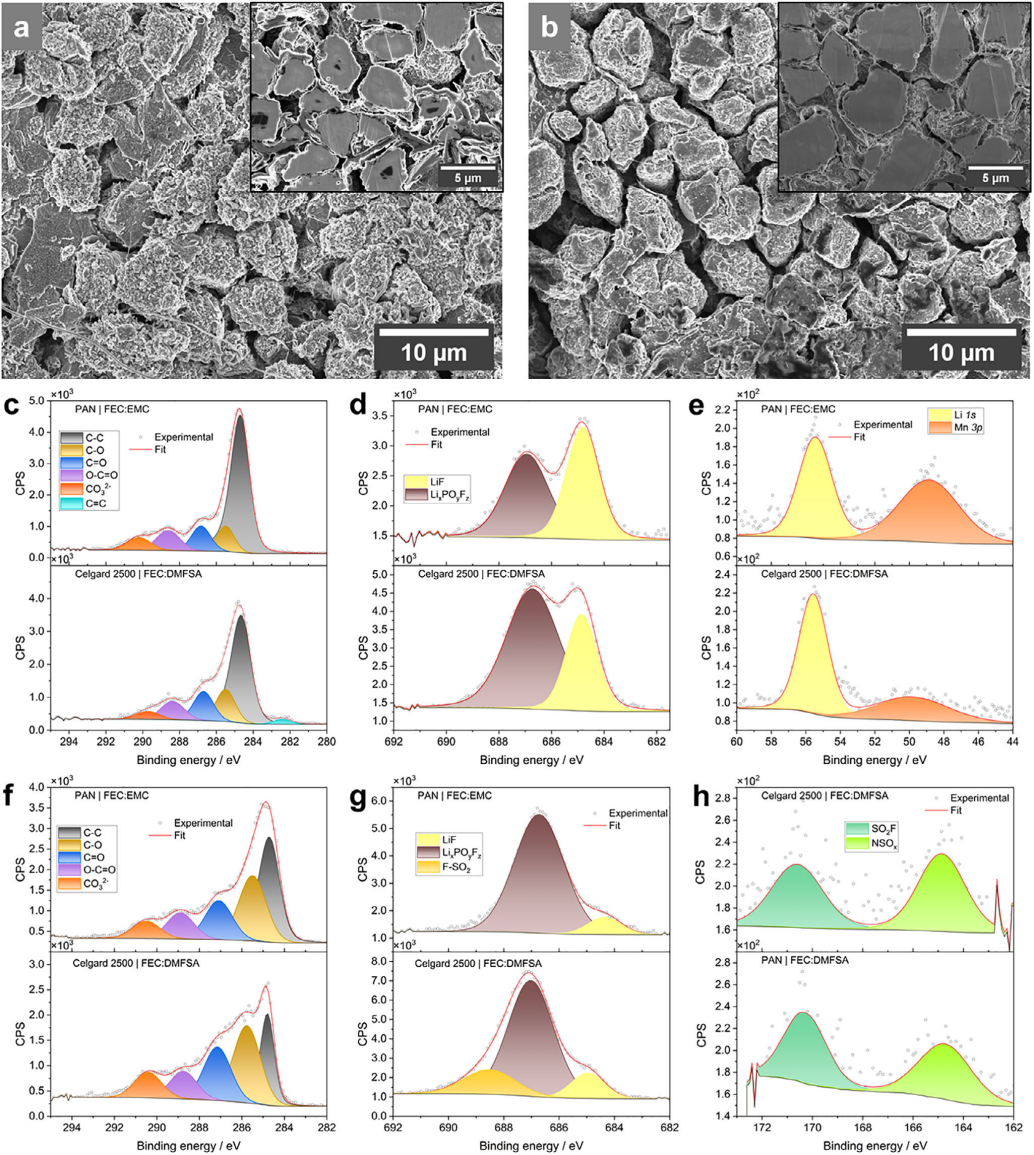
SEM images of the harvested µ‐Si anodes after 50 cycles with (a) 1 m LiPF_6_ in FEC: EMC 3:7 (vol.%) and PAN separator and (b) 1 m LiPF_6_ in FEC: DMFSA 3:7 (vol.%) as electrolyte added to a Celgard 2500 separator. XPS surface chemical analysis showing the (c) C 1s spectra, (d) F 1s spectra and (e) Li 1s spectra of the µ‐Si anodes and (f) C 1s spectra, (g) F 1s spectra and (h) S 2p spectra of the LMR cathodes harvested after 50 cycles.

The morphology of the harvested µ‐Si particles and of the Celgard separators (Figure S14) after 50 cycles shows pronounced heterogeneous surface morphological features in Figure 6a,b while the insets indicate the formation of amorphous Si at the surface with the particle core remaining crystalline [46]. Such behavior is attributed to the unbalanced N/P capacity ratio of 2.27, which prevents complete utilization of the µ‐Si particles active material during the electrochemical reaction, prolonging their cycle life time. Nevertheless, the morphological changes of the harvested µ‐Si particles seem to be more affected by the electrolyte formulation rather than the separator type, as shown also by Figure S13. With FEC: EMC‐based electrolyte, larger agglomerates with rough and highly textured surfaces resembling a cauliflower‐like morphology are covering the surface of the µ‐Si particles (Figure 6a), while a smoother and more consolidated surface coating is observed for the µ‐Si particles harvested from the cells using FEC: DMFSA‐based electrolyte (Figure 6b). Furthermore, with FEC: EMC‐based electrolyte, additional elongated morphological features resembling sub‐micrometer‐sized nodules are noticed on the surface of the µ‐Si particles, especially with a larger size and more nodular deposits for the electrodes cycled with Celgard 2500 separator as seen in Figure S13.

Further insights of the surface chemical composition of these layers were carried out by x‐ray photoelectron spectroscopy (XPS) measurements of the harvested electrodes (Figure 6c–h and Figure S15). The comparison of C 1s core spectra in Figure 6c indicates less differences across the two electrolyte formulations, implying strongly that the C‐species present in the SEI mainly reside from the carbonate‐based solvent decomposition (i.e., FEC and EMC). However, variations in the amount of F‐species are observed in the F *1s* core spectra (Figure 6d) of the µ‐Si electrodes surface harvested from the cells using FEC: EMC‐based electrolyte compared to FEC: DMFSA‐based electrolyte, where a higher amount of LiF species at ≈ 684.8 eV relative to fluorophosphate species (i.e. Li_x_PO_y_F_z_) at ≈ 686.95 eV are present with FEC: EMC‐based electrolyte compared to FEC: DMFSA. Such changes can be further rationalized by the electrochemical reduction behavior of DMFSA, as shown by the cyclic voltammetry measurements (Figure S16a). Two distinct DMFSA reduction processes at approximately 1.07 and 1.44 V vs. Li|Li^+^ are observed, both occurring at significantly higher potentials than EMC and FEC reduction. These additional reduction pathways indicate that DMFSA actively participates in the initial electrolyte decomposition and competes with FEC and EMC during interphase formation, thereby modifying the composition of the resulting SEI. Furthermore, the analysis of the Li *1s* core spectra in Figure 6e reveals around binding energies between 46 to 53 eV, among the LiF presence at ≈ 55.62 eV, additional information related to the process of transition metal deposition. The surface chemical composition of the harvested µ‐Si electrodes shows a higher content of manganese species ≈ 48.9 eV in the Mn *3p* core spectra region with FEC: EMC‐based electrolyte compared to FEC: DMFSA‐based electrolyte. A similar trend is observed independent of the separator used as is presented in Figure S15c. Changes in the surface chemical composition of the LMR electrodes harvested from these cells also indicates in the C *1s* core spectra in Figure 6f a predominant carbonate‐based solvent decomposition, while the F *1s* core spectra in Figure 6g shows the presence of a lesser amount of LiF at the surface of LMR electrodes relative to the fluorophosphates species independent of the electrolyte formulation. Furthermore, the involvement of DMFSA in the CEI formation is indicated by the presence of sulfur and nitrogen species in Figure 6h and Figure S15f. Such S‐containing species can be associated with DMFSA decomposition, resulting in various fragments that were assigned to the sulfonyl group (‐SO_2_F) and some N‐SO_2_ remaining moieties ≈ 170 and ≈ 164.8 eV indicated in the S *2p* and the N *1s* spectra ≈ 400.8 eV in Figure S15f resulted from the DMFSA F‐S bond breaking during the electrochemical reaction. The oxidation behavior observed for the FEC: DMFSA electrolyte formulation (Figure S16b) in the linear sweep voltammogram provides direct electrochemical evidence for such decomposition reactions, where the decomposition products result in sulfur‐ and nitrogen‐rich CEI species. Such species can improve the properties of the CEI and SEI resulting in the observed enhanced electrochemical performance [44, 47].

In summary, despite the fact that the separator morphology influenced the electrochemical performance of the cells using FEC: EMC‐based electrolytes, the interphase chemistry outweighed the separator effect: the cyclic voltammetry results demonstrate that DMFSA participates in the earliest reduction reactions at the electrode surface, while XPS confirms that these reactions generate sulfur‐ and nitrogen‐containing interphase species and reduce transition‐metal deposition on the silicon anode. Together, these results demonstrate that the beneficial interphase chemistry induced by DMFSA outweighs the influence of separator morphology, highlighting the dominant role of electrolyte formulation in determining the electrochemical performance of the LMR||µ‐Si cells.

## 3 Conclusions

The electrochemical performance of LMR||µ‐Si cells is strongly affected by the nature and properties of the separator used to host the liquid electrolyte, moreover through a rational combination of different electrolyte constituents. The effect of separator properties and the electrolyte formulation was demonstrated with FEC: EMC‐based electrolyte, while electrolyte formulations based on FEC: DMFSA solvent mixture, showed improved MacMullin number with both separators, resulting in similar electrochemical performances. The formation of Li metal dendrites on the surface of the µ‐Si electrodes was mainly observed for FEC: EMC‐based electrolyte formulations with polyolefin Celgard 2500 separator, a process that leads to a fast capacity decay after only 60 cycles. Such behavior accounted to a continuous shift toward 0 V vs. Li|Li^+^ of the end of charge potential point of the µ‐Si electrode, while the potential analysis of the 3‐electrode cell data using the electrospun PAN membrane showed negligible potential slippage. Based on the SEM observations, one can also assume that with the electrospun PAN membrane, the PAN nano‐fibers entangle between the µ‐Si particles maintaining contact with the µ‐Si particles/electrode during the electrode breathing process (volume expansion and contraction as a result of Li^+^ ion uptake and release by the µ‐Si active material particle). Furthermore, the capacity retention of the LMR||µ‐Si cells with FEC: DMFSA‐based electrolyte formulation was extended for more than 400 cycles as compared to the FEC: EMC‐based electrolyte counterpart, despite similar contact angle values. Here, the effect of the separator on the electrochemical performance was less evident, suggesting a stronger contribution to the electrochemical behavior of the interphasial chemistry. The XPS measurements indicate the formation of decomposition products derived from the DMFSA solvent at the LMR electrode surface. Owing to its strongly electron‐withdrawing sulfonamide functionality, DMFSA enhances the cathode electrolyte interphase (CEI), promoting the formation of an inorganic‐rich film that effectively suppresses parasitic reactions at high voltages, particularly transition metal dissolution.

## Author Contributions

**Marian Cristian Stan**: conceptualization, investigation, writing – original draft, methodology, writing – review and editing, formal analysis. **Bärbel Arling**: investigation, conceptualization, writing – original draft, methodology, writing – review and editing. **Gerrit Michael Overhoff**: conceptualization, investigation, writing – original draft, methodology, writing – review and editing. **Kirstin Darida**: investigation, methodology. **Lucas Marquart**: investigation, methodology. **Peng Yan**: investigation, formal analysis. **Lara Kersting**: investigation, formal analysis. **Linus Voigt**: investigation, formal analysis, methodology. **Laurin Profanter**: investigation, formal analysis. **Hyung‐Tae Kim**: investigation, formal analysis. **Martin Winter**: funding acquisition, writing – review and editing, supervision, resources, project administration. **Gunther Brunklaus**: project administration, supervision, resources, writing – review and editing. **Isidora‐Cekic Laskovic**: conceptualization, writing – review and editing, funding acquisition, project administration, supervision, resources.

## Conflicts of Interest

The authors declare no conflicts of interest.

## Supporting information




**Supporting File**: advs78391‐sup‐0001‐SuppMat.docx.

## Data Availability

The data that support the findings of this study are available from the corresponding author upon reasonable request.
