# Supplementary material for: Synergistic Effects of Separator Architecture and Electrolyte Chemistry on the Electrochemical Stability of Lithium Ion Batteries With Li–Mn Rich Cathodes and µ‐Si Anodes
Source: Adv Sci (Weinh). 2026 Oct 20:e78391. Online ahead of print. doi: 10.1002/advs.78391 (PMC13683978; doi:10.1002/advs.78391)
Supplement: Supplementary file 1 [file ADVS-9999-e78391-s001.docx]

Supporting Information

Synergistic Effects of Separator Architecture and Electrolyte Chemistry on the Electrochemical Stability of Lithium Ion Batteries with Li–Mn Rich Cathodes and µ-Si Anodes

*Marian Cristian Stan^1,‡^, Bärbel Arling^1,2,‡^, Gerrit Michael Overhoff^1,‡^, Kirstin Darida^1^, Lucas Marquart^1^, Peng Yan^1^, Lara Kersting^3^, Linus Voigt^2,3^, Laurin Profanter^3^, Hyung-Tae Kim^3^, Martin Winter^1,3^, Gunther Brunklaus^1^ and Isidora-Cekic Laskovic^1*^*

*^1^Institute of Energy Materials and Devices, Helmholtz Institute Münster: Ionics in Energy Storage (IMD-4 / HI MS), Forschungszentrum Jülich GmbH, Corrensstraße 48, 48149 Münster, Germany*

*^2^International Graduate School for Battery Chemistry, Characterization, Analysis, Recycling, and Application (BACCARA), University of Münster, Corrensstraße 40, 48149 Münster, Germany*

*^3^MEET Battery Research Center, University of Münster, Corrensstraße 46, 48149 Münster, Germany*

*^4^LG Energy Solution, R&D campus Daejeon, 188 Munji-ro, Yuseong-gu, Daejeon 34122, Korea*

‡ these authors contributed equally

*Corresponding Author*: i.cekic-laskovic@fz-juelich.de*

**Experimental Section**

*Preparation of electrospun PAN membranes:* A solution of 8 wt.% polyacrylonitrile (PAN viscosity-average molecular weight (M_ʋ_) 150.000 g mol^-1^, Sigma Aldrich) in *N,N*-dimethylformamide (DMF, VWR Chemicals, ≥ 99.8%) was prepared by stirring at 60°C for 1 hour. The cooled down solution was then electrospun onto an Aluminum foil covered collector plate using a flow rate of 1.3 ml h^−1^ and an applied voltage of 15 kV between the nozzle (17 Gauge needle) and the collector plate (18 cm distance) using an Inovenso NS24. The electrospinning was carried out for two hours at 25±2% humidity and around 25°C, resulting in a membrane thickness of approximately 60 µm. Following the electrospinning preparation, the electrospun PAN membranes were calendered to a thickness of 20 to 25 µm (porosity of 60-70%, determined based on the measured thickness after calendaring) and sintered to a temperature of 210 °C for 35 minutes under normal atmospheric conditions.[1]

*Contact angle measurements:* Contact angle measurements were carried out using a Krüss Drop Shape Analyzer (DSA) at 20°C and inside a dry room with dew point of −54°C.[2] Every measurement was performed at least 3 times to ensure reproducibility.

*Electrolyte formulation*: The electrolyte 1 M LiPF_6_ in FEC:EMC 3:7 (vol. %) was prepared by mixing fluoroethylene carbonate (FEC, E-Lyte Innovations, battery grade) and ethyl methyl carbonate (EMC, E-Lyte Innovations, battery grade) in a 3:7 ratio by volume followed by the dissolution of the required amount of lithium hexafluorophosphate (LiPF_6_, E-Lyte Innovations, battery grade) in order to reach a 1 M concentration. *N,N*-dimethylsulfamoyl fluoride (DMFSA, TCI, > 98%) was used to prepare the 1 M LiPF_6_ in FEC:DMFSA 3:7 (vol. %) electrolyte formulation using the same preparation method.

*Electrochemical measurements*: Electrochemical cells were assembled using CR 2032 cell setup for 2-electrode and PAT cells (EL-Cell) for 3 electrode measurements (using Li metal as RE electrode) and the electrodes (Li_1.34_Mn_0.65_Ni_0.35_O_2_ cathode denoted as LMR with an area capacity of 5.24 mAh cm^−1^ and 14 mm diameter; microcrystalline Si anode denoted as μ-Si with an area capacity of 11.9 mAh cm^−1^ and 15 mm diameter) that were kindly provided by LG Energy Solution, Ltd.. The constant current constant voltage (CCCV) cycling[3] of the LMR||μ-Si cells was evaluated in the voltage range 4.4-2.5 V using a C/3 rate. Prior cycling, a formation cycle was carried at C/10 rate within the voltage range of 4.65-2.0 V, followed by a second charge/discharge cycle at C/10 in the voltage range of 4.4-2.0 V. Each charge step was followed by a constant voltage step with the limitation in current (*i* < C/20). In all cases the currents were calculated based on the weight and practical capacity of the LMR cathode the active material. The electrolyte amount of 35 μL 1 M LiPF_6_ in FEC:EMC 3:7 (vol. %) was added dropwise to either the PP Celgard 2500 separator (16 mm diameter) or the PAN membrane (16 mm diameter). For the C-rate tests, the current was incrementally increased from 0.05-0.1-0.2-0.33-0.5-0.75-1.0-1.5-2.0-3.0-5.0C for 5 cycles at each C-rate. The C-rate current was calculated based on the cathode active material loading on each electrode. Galvanostatic intermittent titration technique (GITT) tests were carried out in 3-electrode cell configurations by applying successive current pulses followed by relaxation periods until the cell voltage reached the upper cutoff value of 4.65 V and the lower voltage cutoff value of 2.0 V. The current pulses were applied using a current of C/10 for 10 minutes followed by relaxation time between the current pulses of 2 hours. The measurements were carried out using a battery testing device Maccor 4000 series (Maccor) and a VMP 3 (BioLogic) equipped with a climatic chamber with a constant temperature of 20°C. For the determination of the MacMullin number, the separator and the electrolyte were assembled in a 2-electrode CR2032 cell using two polished SUS 316 spacers (1 mm) and a 1.5 mm spring. An amount of 20 µl electrolyte was added on both sides of the separators and the AC impedance measurement was carried out in the frequency range of 1 MHz to 100 Hz with a voltage amplitude of 10 mV using the VMP 3 (BioLogic). Before the measurement was started, the cell was allowed to equilibrate in the climatic chamber at 20 °C for ca. 1 hour. The results were fitted using an equivalent circuit composed of a resistor (R) in series with a constant phase element (CPE). The method measurement of the ionic conductivity of the electrolytes employed here was reported elsewhere.[4, 5]

*SEM and EDX:* The morphological changes of the harvested μ-Si electrodes from the LMR||μ-Si 3-electrode cells were investigated by means of scanning electron microscope (SEM, Carl-Zeiss CrossBeam 550) using an acceleration voltage of 3 kV and a working distance (WD) of 5 mm. The elemental composition of the materials surface and Li detection was determined using the energy-dispersive X-ray spectroscopy (EDX) at an SEM accelerating voltage of 5 kV, image magnification of 5000 x, WD of 8.5 mm and a current of 1 nA by employing a windowless Ultim Extreme detector (Oxford Instruments) and the AZtec software (Oxford Instruments, version 4.4.7495.1). Cross-sections of the harvested electrodes were prepared using an Cryo-Cross Polisher (IB-19520CCP, JEOL).

*X-ray Photoelectron Spectroscopy (XPS):* To carry out the X-ray photoelectron spectroscopy (XPS) measurements, the harvested electrodes were recovered inside a glove-box under Ar atmosphere from the cycled cells and carefully washed with EMC to remove the remaining salt prior drying under reduced pressure. After that the samples were mounted on a sample holder and transferred under inert atmosphere to an Axis Ultra DLD XPS (Kratos Analytical) where the samples were held under ultrahigh vacuum (<10^-8^ mbar) for 12 hours to remove volatile species. Afterwards, the core and survey spectra were recorded out using a monochromatic Al Kα source (hν = 1486.6 eV) at an emission current of 10 mA with an accelerating voltage of 12 kV and an angle of emission of 0 °. Positive charging of the samples surface was suppressed using a charge neutralizer. Core spectra were recorded for C *1s*, Li *1s*, O *1s*, N *1s*, F *1s*, P *2p*, S *2p*, Ni *2p* and Mn *2p* with a pass energy of 20 eV and survey spectra were recorded with a pass energy of 160 eV. To confirm for reproducibility, the XPS measurements were carried out on two different electrodes using 5 iterations per spot. CasaXPS software (Version 2.3.26PR1.0) was used for XPS data analysis with the binding energy (BE) of all the core spectra referenced to the sp^3^-component of the hydrocarbon signal C *1s* centered at 284.8 eV.[6, 7] To account for inelastically scattered photoelectrons, a Shirley-type background was used for the fitting. All peaks were fitted with an LA(2,2,250) line shape except of the adventitious carbon peak where the line shape LF(0.75,1,200,500) was used.[7] One representative measurement is displayed.

Figure S1. Zoom in of the Nyquist plot for the separators soaked with 20 µl of electrolyte between 2 polished SUS316 plates (16 mm diam.).

Table S1. Parameters used for the calculation of the MacMullin numbers with FEC:EMC-based electrolytes.

| **Separator** | **Thickness**  **µm** | **R_ion_**  **Ω** | **σ_El.*_**  **mS cm^−1^** | **σ_Sep.El._**  **S cm^−1^** | **N_M_** |
| --- | --- | --- | --- | --- | --- |
| Celgard 2500 | 22.33 | 3.63 | 7.455 | 3.13 × 10^−4^ | 23.8* |
| PAN | 21 | 1.31 | 7.455 | 8.17 × 10^−4^ | 9.1 |
| *Electrolyte: 1 M LiPF_6_ in FEC:EMC 3:7 (vol. %)  N_M_: MacMullin number | | | | | |

Figure S2. Comparison of the extended cycling of the LMR||µ-Si cells until EOL is reached (1000 cycles) with 1 M LiPF_6_ in FEC:EMC 3:7 (vol. %) as electrolyte using the Celgard 2500 (dark gray) and electrospun PAN (red) membrane.

Figure S3. Evolution of the specific discharge capacities from LMR||µ-Si cells with 3-electode configuration using Celgard 2500 and electrospun PAN membrane and 1 M LiPF_6_ in FEC:EMC 3:7 (vol. %) as electrolyte at 20°C.

Figure S4. SEM images of the cycled µ-Si electrodes harvested from the 2-electrode LMR||µ-Si cells after 1000 cycles with (a) Celgard 2500 separator and (b) electrospun PAN membrane.

Figure S5. Li-EDX of the harvested µ-Si electrodes from the 2-electrode LMR||µ-Si cells after 1000 cycles.

Figure S6. Evolution of the Ohmic drop from the GITT measurements of the first two cycles of an LMR||µ-Si 3-electrode cell with Celgard 2500 and electrospun PAN membrane with 1 M LiPF_6_ in FEC:EMC 3:7 (vol. %) at 20°C for the (a) pulse step and (b) relaxation step.

Figure S7. SEM cross-section images of Celgard 2500 separator (a) before and (b) after 152 cycles harvested from the LMR||µ-Si cells with 1 M LiPF6 in FEC:EMC 3:7 (vol. %).

Figure S8. SEM morphological investigations of the Celgard 2500 separator harvested from the LMR||µ-Si cells after 152 cycles showing (a-b) cross-section image of the separator with remaining µ-Si particles, (c) top-view of the separator surface with the inset showing a digital image of the harvested Celgard 2500 separator, and (d) surface EDX spectra of the harvested Celgard 2500 separator with the inset showing an enlarged SEM cross-section image indicating the surface SEI layer.

Figure S9. SEM images of the µ-Si electrode surface after 152 cycles showing (a) remaining SEI attached to the µ-Si particles and (b) SEI fragments. In both images, the morphology of the Celgard 2500 surface can be recognized on these structures.

Figure S10. Comparison of the accumulated Coulombic inefficiency (ACI) evolution for the LMR||µ-Si cells assembled using the electrospun PAN membrane and 1 M LiPF_6_ in FEC:EMC 3:7 (vol. %) and 1 M LiPF_6_ in FEC:DMFSA 3:7 (vol. %) as electrolyte solution.

Figure S11. Morphologies of electrodes harvested from the LMR||μ-Si cells after C-rate investigations using Celgard 2500 and electrospun PAN membranes with 1 M LiPF_6_ in FEC:EMC 3:7 (vol. %) as electrolyte: (a) µ-Si - Celgard 2500; (b) µ-Si – PAN; (c) LMR - Celgard 2500 and (d) LMR – PAN.

Figure S12. Contact angle measurements of the (a) Celgard 2500 separators and (b) electrospun PAN membrane using 1 M LiPF_6_ in FEC:DMFSA 3:7 (vol. %) as electrolyte. (c) Zoom of the Nyquist plot of the separators soaked with 20 µl of 1 M LiPF_6_ in FEC:DMFSA 3:7 (vol. %) as electrolyte between 2 polished SUS316 plates (16 mm diam.)

Table S2. Parameters used for MacMullin numbers calculations of separators with FEC:DMFSA-based electrolytes.

| **Separator** | **Thickness**  **µm** | **R_ion_**  **Ω** | **σ**_El.*_**  **mS cm^−1^** | **σ_Sep.El._**  **S cm^−1^** | **N_M_** |
| --- | --- | --- | --- | --- | --- |
| Celgard 2500 | 23.26 | 2.44 | 4.572 | 4.87 × 10^−4^ | 9.38 |
| PAN | 21 | 1.43 | 4.572 | 7.49 × 10^−4^ | 6.10 |
| *Electrolyte: 1 M LiPF_6_ in FEC:DMFSA 3:7 (vol. %)  **Ionic conductivity value at 20 °C  N_M_: MacMullin number | | | | | |

Figure S13. SEM images of the harvested μ-Si anodes after 50 cycles with (a) 1 M LiPF_6_ in FEC:EMC 3:7 (vol. %) and Celgard 2500 separator and (b) 1 M LiPF_6_ in FEC:DMFSA 3:7 (vol. %) as electrolyte added to a PAN electrospun membrane.

Figure S14. SEM top-view morphology and digital images of the Celgard 2500 separators harvested from LMR||µ-Si cells after 52 cycles using (a) FEC:EMC and (b) FEC:DMFSA-based electrolyte formulations. The corresponding EDX spectra with the inset showing an enlarged region of the surface cross-section of the Celgard 2500 separators harvested from LMR||µ-Si cells after 52 cycles using (c) FEC:EMC and (d) FEC:DMFSA-based electrolyte formulations.

Figure S15. XPS surface chemical analysis showing the (a) C 1s spectra, (b) F 1s spectra and (c) Li 1s spectra of the μ-Si anodes and (d) C 1s spectra, (e) F 1s spectra and (f) N 1s spectra of the LMR cathodes harvested after 50 cycles from the cells with Celgard 2500 separator and FEC:EMC-based electrolyte and PAN membrane with FEC:DMFSA-based electrolyte.

Figure S16. Comparison of (a) cathodic electrochemical stability of 1 M LiPF_6_ in FEC:EMC 3:7 (vol. %), 1 M LiPF_6_ in FEC:DMFSA 3:7 (vol. %) and 1 M LiPF_6_ in DMFSA:EMC 3:7 (vol. %) electrolytes determined in 3-electrode cells using cyclic voltammetry on μ-Si electrodes and (b) linear sweep voltammetry of 1 M LiPF_6_ in FEC:EMC 3:7 (vol. %) and 1 M LiPF_6_ in FEC:DMFSA 3:7 (vol. %) on C-coated Al foil to 5 V vs. Li|Li^+^. All measurements were carried out at 20 °C.

References

References

1	S. H. Kang, J.-K. Jang, H. Y. Jeong, et al. "Polyacrylonitrile/Phosphazene Composite-Based Heat-Resistant and Flame-Retardant Separators for Safe Lithium-Ion Batteries.", *ACS Applied Energy Materials* (2022), *5* (2), 2452–2461, https://doi.org/10.1021/acsaem.1c03948.

2	P. Schmitz, M. Kolek, D. Diddens, et al. "Counterintuitive trends of the wetting behavior of ionic liquid-based electrolytes on modified lithium electrodes.", *Physical chemistry chemical physics : PCCP* (2017), *19* (29), 19178–19187, https://doi.org/10.1039/c7cp03716c.

3	X. Liu, M. Gao, J. Zhao, et al. "Effects of charging protocols on the cycling performance for high-energy lithium-ion batteries using a graphite-SiOx composite anode and Li-rich layered oxide cathode.", *Journal of Power Sources* (2021), *495*, 229793, https://doi.org/10.1016/j.jpowsour.2021.229793.

4	A. Narayanan Krishnamoorthy, C. Wölke, D. Diddens, et al. "Data‐Driven Analysis of High‐Throughput Experiments on Liquid Battery Electrolyte Formulations: Unraveling the Impact of Composition on Conductivity**.", *Chemistry–Methods* (2022), *2* (9), https://doi.org/10.1002/cmtd.202200008.

5	M. C. Stan, P. Yan, G. M. Overhoff, et al. "Unraveling Influential Factors of Stainless-Steel Dissolution in High-Energy Lithium Ion Batteries with LiFSI-Based Electrolytes.", *ChemElectroChem* (2025), *12* (6), e202400632, https://doi.org/10.1002/celc.202400632.

6	N. Fairley, V. Fernandez, M. Richard‐Plouet, et al. "Systematic and collaborative approach to problem solving using X-ray photoelectron spectroscopy.", *Applied Surface Science Advances* (2021), *5*, 100112, https://doi.org/10.1016/j.apsadv.2021.100112.

7	B. Heidrich, M. Börner, M. Winter, P. Niehoff. "Quantitative determination of solid electrolyte interphase and cathode electrolyte interphase homogeneity in multi-layer lithium ion cells.", *Journal of Energy Storage* (2021), *44*, 103208, https://doi.org/10.1016/j.est.2021.103208.
